# Aag DNA Glycosylase Promotes Alkylation-Induced Tissue Damage Mediated by Parp1

**DOI:** 10.1371/journal.pgen.1003413

**Published:** 2013-04-04

**Authors:** Jennifer A. Calvo, Catherine A. Moroski-Erkul, Annabelle Lake, Lindsey W. Eichinger, Dharini Shah, Iny Jhun, Prajit Limsirichai, Roderick T. Bronson, David C. Christiani, Lisiane B. Meira, Leona D. Samson

**Affiliations:** 1 Department of Biological Engineering, Massachusetts Institute of Technology, Cambridge, Massachusetts, United States of America; 2 Center for Environmental Health Sciences, Massachusetts Institute of Technology, Cambridge, Massachusetts, United States of America; 3 Faculty of Health and Medical Sciences, University of Surrey, Guildford, United Kingdom; 4 Department of Pathology, Harvard Medical School, Cambridge, Massachusetts, United States of America; 5 Department of Environmental Health, Harvard School of Public Health, Boston, Massachusetts, United States of America; 6 Department of Biology, Massachusetts Institute of Technology, Cambridge, Massachusetts, United States of America; 7 The David H. Koch Institute for Integrative Cancer Research, Massachusetts Institute of Technology, Cambridge, Massachusetts, United States of America; National Institute of Environmental Health Sciences, United States of America

## Abstract

Alkylating agents comprise a major class of front-line cancer chemotherapeutic compounds, and while these agents effectively kill tumor cells, they also damage healthy tissues. Although base excision repair (BER) is essential in repairing DNA alkylation damage, under certain conditions, initiation of BER can be detrimental. Here we illustrate that the alkyladenine DNA glycosylase (AAG) mediates alkylation-induced tissue damage and whole-animal lethality following exposure to alkylating agents. Aag-dependent tissue damage, as observed in cerebellar granule cells, splenocytes, thymocytes, bone marrow cells, pancreatic β-cells, and retinal photoreceptor cells, was detected in wild-type mice, exacerbated in *Aag* transgenic mice, and completely suppressed in *Aag* ^−/−^ mice. Additional genetic experiments dissected the effects of modulating both BER and Parp1 on alkylation sensitivity in mice and determined that Aag acts upstream of Parp1 in alkylation-induced tissue damage; in fact, cytotoxicity in WT and *Aag* transgenic mice was abrogated in the absence of Parp1. These results provide *in vivo* evidence that Aag-initiated BER may play a critical role in determining the side-effects of alkylating agent chemotherapies and that Parp1 plays a crucial role in Aag-mediated tissue damage.

## Author Summary

Alkylating agents are genotoxic chemicals that induce both toxic and mutagenic DNA damage through addition of an alkyl group to DNA. Alkylating agents are routinely and successfully used as chemotherapeutic therapies for cancer patients, with one major disadvantage being the significant toxicity induced in non-tumor tissues. Accordingly, identifying factors that modify susceptibility to alkylation-induced toxicity will provide valuable information in designing cancer therapeutic regimens. This study used mouse genetic experiments to investigate whether proteins important in the base excision repair pathway modulate susceptibility to alkylating agents. In addition to whole-animal toxicity at high doses, treatment of mice with alkylating agents resulted in severe damage to numerous tissues including the cerebellum, retina, bone marrow, spleen, thymus, and the pancreas. We illustrate that the DNA glycosylase Aag can actually confer, rather than prevent, alkylation sensitivity at both the whole-animal and tissue level; i.e., *Aag* transgenic animals are more susceptible than wild type, whereas *Aag*-deficient animals are less susceptible than wild type to alkylation-induced toxicity. Further genetic experiments show that the Aag-mediated alkylation sensitivity is dependent on Parp1. Given that we observe a wide range of human AAG expression among healthy individuals, this and other base excision repair proteins may be important factors modulating alkylation susceptibility.

## Introduction

DNA damage continually arises from environmental agents and reactive byproducts of normal cellular function. Moreover, DNA damage is deliberately induced during the course of cancer chemotherapy. Such damage can result in cell death, mutagenesis, and genetic instability thus promoting tissue degeneration, aging, cancer, and sometimes death. DNA repair pathways have evolved to cope with recurring DNA damage, providing protection against carcinogenesis, neurodegeneration, and premature aging [1]–[5]. Understandably, loss of function mutations have been extensively studied, whereas genetic variants that result in increased DNA repair activity have not received the same attention, primarily because decreased DNA repair is thought to be more relevant for increased cancer risk. While this concept is accurate for many DNA repair proteins [1]–[3], a growing body of evidence suggests that increased levels of certain DNA repair enzymes can result in loss of coordination between the enzymatic steps within a particular DNA repair pathway; such loss of coordination can negatively impact cellular homeostasis [6]–[9].

**Figure 1:**
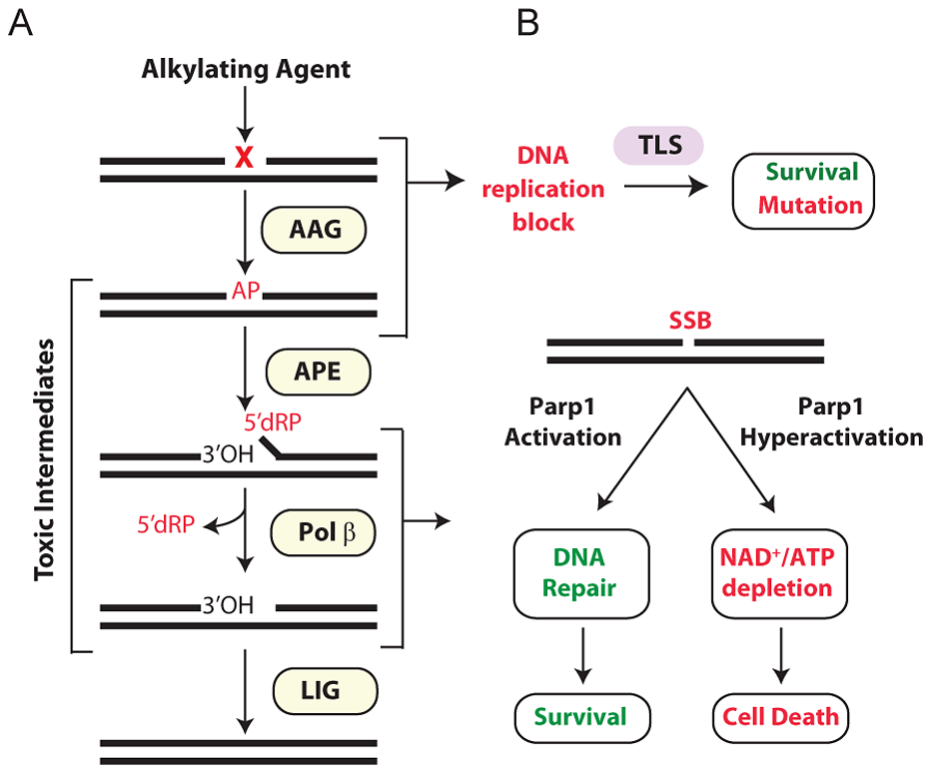
Cellular processing and repair of DNA base lesions in DNA. (A) DNA base lesions induced by S_N_1 or S_N_2 alkylating agent are recognized and excised by the DNA glycosylase, Aag, to generate an AP site. The base excision repair (BER) pathway continues when an AP endonuclease cleaves the DNA backbone to generate 3′ hydroxyl and 5′ deoxyribose phosphate (5′dRP) termini. Polymerase β removes the 5′dRP species, and inserts the missing DNA bases; DNA ligase completes BER by sealing the nicked DNA. (B) Error-prone translesion (TLS) polymerases can assist in the tolerance or bypass of base lesions and AP sites. Parp1 has an important role in regulating the response to DNA damage. During times of moderate DNA damage, Parp1 activation facilitates BER. Upon high levels of DNA damage, Parp1 undergoes hyperactivation; cells consequently suffer NAD^+^/ATP depletion, triggering cell death.

The base excision repair (BER) pathway acts on a wide range of DNA base lesions including alkylated, oxidized, and deaminated bases, as well as abasic (AP) sites and DNA single-strand breaks (SSBs) (reviewed in [6], [10]). In its most simplified form, BER is coordinated into 4 main steps (Figure 1A). DNA glycosylases recognize and excise specific base lesions by cleaving the N-glycosyl bond, forming an AP site. AP endonuclease (APE1) then hydrolyzes the phosphodiester backbone, generating a single-stranded DNA break (SSB) with 3′OH and 5′deoxyribose-5-phosphate (5′dRP) termini. DNA polymerase β (Pol β) contains a lyase domain that removes the 5′dRP terminus and a polymerase domain that replaces the missing nucleotide. Finally, BER is completed upon ligation of the nick by DNA Ligase I or the Xrcc1/Ligase IIIα complex (Figure 1A).

Importantly, numerous BER intermediates (AP sites, 5′dRP termini, and SSBs) are toxic if allowed to accumulate rather than being efficiently shuttled through the downstream BER steps (Figure 1A). Both SSBs and AP sites exert their toxicity as a function of blocking transcription and replication [11]. Further, large numbers of SSBs can indirectly induce toxicity through the hyperactivation of poly(ADP-ribose) polymerase 1 (Parp1) [12] (Figure 1B). AP sites can also be mutagenic; although translesion DNA polymerases can prevent toxicity by bypassing AP sites, such bypass can generate point mutations [13]–[17]. The 5′dRP intermediate is particularly toxic in mouse embryonic fibroblasts (MEFs) and the alkylation sensitivity of *Polβ* deficient MEFs is almost completely suppressed upon expression of the Pol β 5′dRP lyase domain [18]. The toxic nature of BER intermediates underscores why this pathway must be tightly regulated and why alterations in any step of the pathway, without compensatory changes in upstream/downstream steps, can result in the accumulation of toxic intermediates. A clear example of this was illustrated by the fact that hypersensitivity to the alkylating agent methyl methanesulfonate (MMS) in *Polβ* ^−/−^ MEFs is completely suppressed if BER is not initiated by the alkyladenine DNA glycosylase (AAG, also known as MPG, ANPG) [19]. Therefore, although BER is essential for the repair of many different types of DNA damage, it must be carefully regulated to avoid the accumulation of toxic BER intermediates.

Aag has a wide substrate specificity, excising numerous structurally-diverse lesions, some of which are innocuous (e.g. 7-methylguanine), while others can be replication-blocking and cytotoxic (e.g. 3-methyladenine) [20]–[27]. The absence of Aag should therefore result in unrepaired alkylated DNA bases that are replication-blocking lesions, thus increasing cytotoxicity; strikingly, the converse is seen in certain *Aag* deficient tissues. *Aag^−/−^* bone marrow cells are MMS resistant in *ex vivo* survival assays [28], and *Aag^−/−^* retinal photoreceptor cells are remarkably refractory to MMS-induced death [29]. Thus, when BER is not initiated, MMS-induced cytotoxicity is avoided, presumably by preventing the accumulation of toxic intermediates, and by translesion DNA synthesis (TLS) bypassing lesions in replicating cells (Figure 1B).

The multi-functional protein, Parp1, mediates several cellular processes including stress responses, transcriptional regulation, and DNA SSB repair and BER [30]–[32]. Parp1's role as a molecular sensor of SSBs is well established; upon binding DNA breaks, Parp1 adds poly(ADP-ribose) (PAR) polymers to numerous nuclear proteins including itself, DNA polymerases, DNA ligases, transcription factors, and histones [31], [33]. Parp1 automodification facilitates BER by recruiting the scaffold protein XRCC1 that in turn facilitates the formation of a BER repair complex comprising APE1, DNA Pol β, and DNA ligase III [34]–[36]. Further, PARylation of histones, Parp1, and chromatin remodeling enzymes relaxes chromatin allowing DNA repair proteins access to damaged DNA [37]–[39]. Importantly, Parp1 is also a cell death mediator [12]; upon excessive levels of DNA damage, Parp1 hyperactivation vastly increases NAD^+^ consumption resulting in depletion of both NAD^+^ and ATP, such that cells succumb to bioenergetic failure (Figure 1B). Independent of NAD^+^/ATP depletion, the PAR polymer can also stimulate cell death by facilitating translocation of apoptosis inducing factor (AIF) from mitochondria to the nucleus, resulting in chromatin condensation, caspase-independent DNA degradation, and ultimately cell death [12], [40], [41]. While the various roles of Parp1 in programmed necrosis are still being elucidated, it is quite clear that Parp1 is a central player.

Imbalanced BER can arise either by increased DNA glycosylase activity, or by a decrease in any downstream BER step (reviewed in [6]). For example, decreased Pol β activity, as observed in the *Polβ^Y265C/Y265C^* knock-in mice, results in an accumulation of BER intermediates, causing severe physiological consequences [42]. Interestingly, recent studies generated imbalanced BER by both increasing Aag activity and eliminating Pol β activity; such cells displayed enhanced alkylation sensitivity [43], [44]. Although BER imbalance increases alkylation sensitivity in cultured cells, the effects of BER imbalance on *in vivo* alkylation sensitivity have not yet been extensively studied. Using transgenic mice exhibiting modestly increased Aag activity, we investigated the effects of imbalanced BER in many tissues. We show that *AagTg* mice exhibit dramatic alkylation sensitivity, at both the tissue and the whole-body level, consistent with imbalanced BER leading to the accumulation of toxic intermediates. Moreover, we show that *Parp1* deficiency prevents alkylation-induced damage in numerous tissues, indicating that the Aag-dependent alkylation sensitivity observed *in vivo* occurs in a Parp1-dependent manner.

## Results

### Generation of *AagTg* mice to model BER imbalance *in vivo*

**Figure 2:**
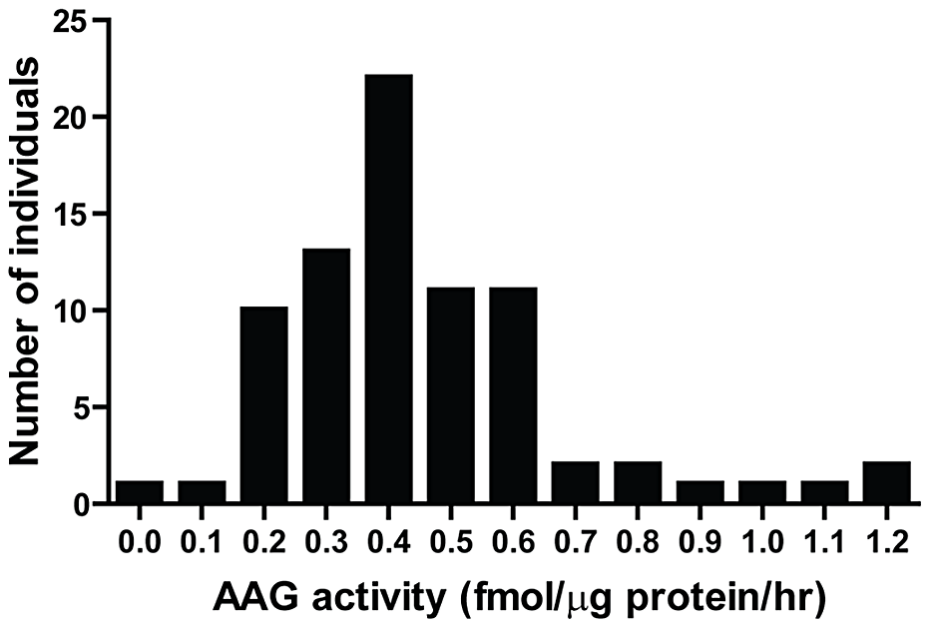
Human peripheral blood mononuclear cells (PBMCs) exhibit a wide range in AAG activity. An *in vitro* glycosylase assay determined AAG activity in PBMCs isolated from 80 healthy individuals.

BER modulation has recently attracted attention as a way to potentiate alkylation sensitivity [6], [30], [44]–[48]. To investigate the consequences of imbalanced BER *in vivo*, we generated *Aag* transgenic (*AagTg*) mice; Table S1 displays the Aag activity levels in three transgenic founder (Fo) lines. Fo line 243 exhibits increased Aag activity in all tissues examined, with a ∼2–9-fold increase compared to WT levels (Table S1 and Figure S1A). Fo line 8756 displays negligible increases in Aag activity in every tissue except the cerebellum. Finally, Aag activity in Fo line 943 tissues falls in-between, with 1.5–4-fold increases compared to WT levels. To add context to the range of Aag activities in our *AagTg* mice, we examined human AAG activity in peripheral blood mononuclear cells (PBMC) of healthy individuals. We observe >10-fold variation in AAG activity in this healthy population, as measured by excision of 1′*N* ^6^ ethenoadenine (εA) bases from DNA (Figure 2); εA represents one of AAG's many substrates [23], [26]. This wide range of AAG activity among healthy individuals is similar to that recently reported [7].

### Imbalanced BER increases whole-body sensitivity to alkylating agents

*AagTg* mice are viable and fertile, and an aging study revealed no apparent differences in lifespan or tumor incidence between WT, *Aag^−/−^* and *AagTg* Fo line 243 (Figure S1B and data not shown). Although increased Aag activity *in vivo* does not significantly alter longevity or spontaneous tumor incidence, it does profoundly affect how mice respond to DNA damage. Similar to our previous published findings, WT and *Aag^−/−^* mice display the same approximate LD_50_ for MMS (150 mg/kg) (Table 1) [28]. However, *AagTg* Fo 243, with a 2–9-fold increase in Aag activity, exhibits a dramatic increase in MMS sensitivity (Table 1). Mice with intermediate Aag levels (*AagTg* Fo 943) have an intermediate LD_50_, and *AagTg* Fo 8756, with negligible Aag activity in most tissues, exhibits the same MMS LD_50_ as WT and *Aag* ^−/−^ mice (Table 1). Therefore, increased Aag activity sensitizes animals to MMS-induced whole-body lethality.

**Table 1:** Approximate MMS LD_50_ for *Aag* transgenic mice.

Mouse Strain	MMS LD_50_
C57Bl/6	150 mg/kg
*Aag^−/−^*	150 mg/kg
*AagTg* Fo 8756*	150 mg/kg
*AagTg* Fo 943*	120 mg/kg
*AagTg* Fo 243*	80 mg/kg

* Indicates that the *Aag* transgene is expressed in an *Aag^−/−^* background.

We next determined whether Aag activity affects the approximate LD_50_ for other genotoxic agents: N-methyl-N-nitrosourea (MNU), azoxymethane (AOM), mitomycin C (MMC), and chloroacetaldehyde (CAA). Table 2 illustrates that while the *AagTg* Fo 243 mice show dramatically increased whole-body sensitivity to three different methylating agents (MMS, MNU and AOM), they were not sensitized to non-methylating genotoxic agents (MMC and CAA). Thus, Aag activity dictates sensitivity to both S_N_1 (MNU and AOM) and S_N_2 methylating agents (MMS), but not to the other genotoxic agents examined. Since the increased Aag activity in *AagTg* Fo 243 mice falls within the range observed in PBMCs of a healthy human population, this founder line was chosen to further examine the consequences of BER imbalance, and are henceforth referred to as *AagTg* mice.

**Table 2:** Approximate LD_50_ of *Aag* ^−/−^ and *Aag* transgenic mice to various genotoxic agents.

	Approximate LD_50_				
Mouse Strain	MMS	MNU	AOM	MMC	CAA
C57Bl/6	150 mg/kg	118 mg/kg	28 mg/kg	9 mg/kg	15.2 mg/kg
*Aag* ^−/−^	150 mg/kg	118 mg/kg	28 mg/kg	9 mg/kg	15.2 mg/kg
*AagTg* Fo 243*	80 mg/kg	35 mg/kg	18 mg/kg	9 mg/kg	15.2 mg/kg

MMS, Methyl methanesulfonate; MNU, N-methyl-N-nitrosourea; AOM, Azoxymethane; MMC, Mitomycin C; CAA, Chloroacetaldehyde.

* Indicates that the *Aag* transgene is expressed in an *Aag^−/−^* background.

### *AagTg* mice exhibit increased MMS cytotoxicity in numerous, but not all, tissues

**Figure 3:**
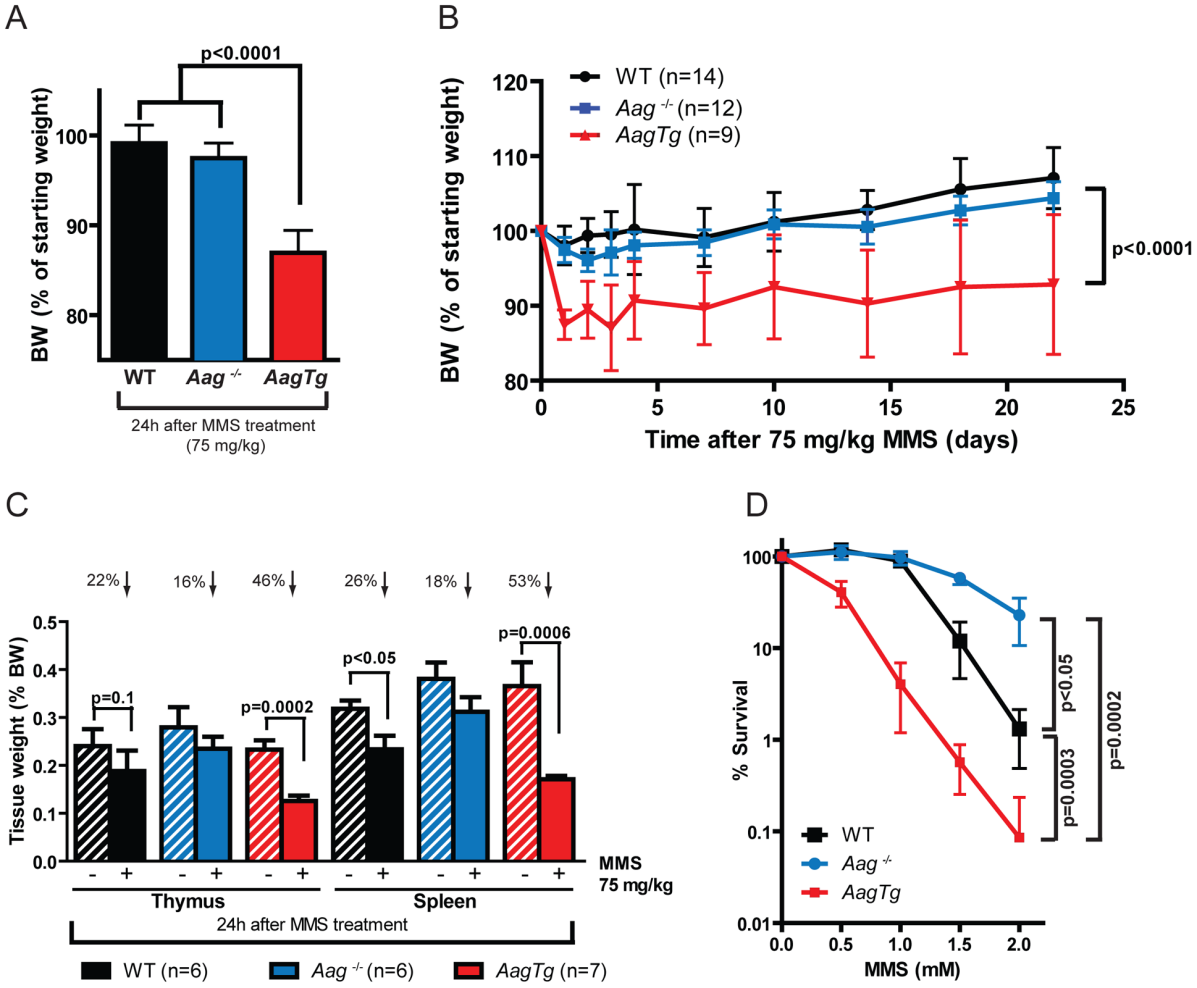
*AagTg* mice are more susceptible to MMS-induced toxicity. (A) Body weight (BW) of WT (n = 14), *Aag* ^−/−^ (n = 12) and *AagTg* (n = 12) mice 24 h following MMS treatment (75 mg/kg). Representative data (mean ± standard deviation) from 3 independent experiments are shown. (B) BW is illustrated for WT (n = 14), *Aag* ^−/−^ (n = 12) and *AagTg* (n = 9) mice following MMS treatment (75 mg/kg). Data represent mean ± standard deviation. (C) Tissue weights of spleen and thymus are illustrated for n>6 per genotype. Striped bars represent untreated tissue weights and solid bars represent tissue weights 24 h following MMS treatment (75 mg/kg). Percent decrease in tissue weight observed following MMS treatment is shown above bars. Data represent mean ± SEM. (D) *Ex vivo* bone marrow (BM) clonogenic survival assays were performed using BM isolated from WT (n = 3), *Aag* ^−/−^ (n = 3) and *AagTg* (n = 3). Data represent mean ± SEM. All the mice used in this figure are males on a pure C57BL/6 background.

Histopathological analysis was performed on tissues harvested from WT, *Aag^−/−^*, and *AagTg* mice 24 h following MMS treatment (150 mg/kg). Because massive cell death was observed in rapidly-proliferating tissues including the spleen, thymus, and bone marrow (BM) for all genotypes, we reduced the MMS dose to 75 mg/kg to better discern any differences in sensitivity in these tissues. Even with this reduced MMS dose, *AagTg* mice displayed evidence of whole-body toxicity whereas WT and *Aag* ^−/−^ mice did not. Remarkably, as early as 24 h following MMS treatment, *AagTg* mice exhibit greater reductions in body weight than WT or *Aag* ^−/−^ mice (Figure 3A), losing >10% of their BW by 24 h; the decreased body weight remains significantly greater than that for WT and *Aag* ^−/−^ mice for over 3 weeks (Figure 3B). Moreover, 24 h following MMS treatment (75 mg/kg), we observe gross tissue atrophy in the thymus and spleen in *AagTg* mice; *AagTg* mice exhibit 46% and 53% decreases in thymus and spleen weight, respectively, compared to untreated tissues (Figure 3C). WT mice also exhibit a slight (26%) but significant decrease in spleen weight following MMS treatment but no evidence of thymic atrophy (compared to untreated WT mice). Strikingly, *Aag* ^−/−^ mice are completely protected from the MMS-induced atrophy in both the thymus and spleen (Figure 3C). Further, *ex vivo* clonogenic survival assays illustrate that *AagTg* bone marrow (BM) cells display dramatically increased MMS sensitivity, compared to WT and *Aag* ^−/−^ mice; as previously published, *Aag^−/−^* BM cells are less sensitive than WT BM cells to MMS (Figure 3D) [28]. However, not all tissues that exhibit increased Aag activity reveal evidence of gross tissue atrophy. Figure S2 illustrates that tissue weights of the heart, kidney, brain, gonadal fat pad, skeletal muscle and liver remain unchanged following MMS treatment (75 mg/kg). Finally, Figure S3 illustrates that *AagTg* mice exhibit severe cell death within the pancreatic β-islets following MMS treatment (150 mg/kg), which is not observed in WT or *Aag^−/−^* mice. The β-cells exhibit nuclear fragmentation and pyknosis, a state of increased chromatin condensation. Taken together, these results reveal that the ∼2–9 fold increase in Aag activity in the thymus, spleen, BM, and pancreas relative to WT (Table S1) renders these tissues dramatically more sensitive to the toxic effects of MMS. Further, we observe increased MMS toxicity in only a subset of tissues expressing the Aag transgene, underscoring the importance of cellular context in determining MMS sensitivity in *AagTg* mice.

**Figure 4:**
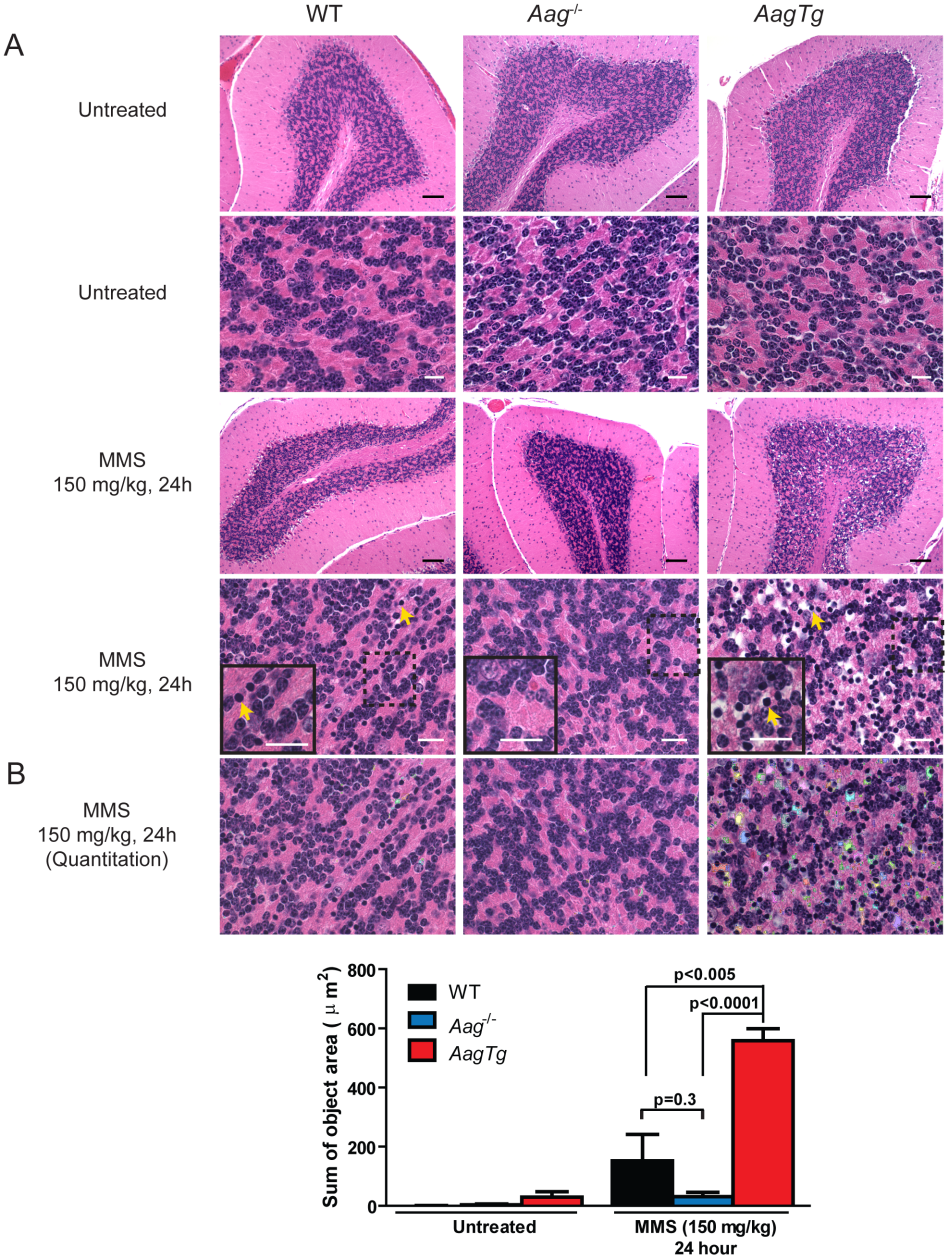
MMS induces severe cerebellar lesions *AagTg* mice. (A) H&E stained image of cerebellar granule cells from WT, *Aag* ^−/−^ and *AagTg* mice either in untreated conditions or 24 h following MMS treatment (150 mg/kg). Representative images are shown of n>6 experiments. Yellow arrows indicate pyknotic nuclei. Scale bar is 100 µm on low magnification images (black bar) and 15 µm on high-magnification images (white bar). Insets contain magnified images of area in dashed boxes. (B) Quantitation of cerebellar phenotype was performed on images from WT (n = 4), *Aag* ^−/−^ (n = 3) and *AagTg* mice (n = 4). Representative images with identified objects (edema) colorized for visualization. Greater than 3 images/cerebella were quantitated per mouse, and the average sum of object area per image is presented. Data represent mean ± SEM. All the mice in this figure are on a pure C57BL/6 background.

Unexpectedly, 24 h following a high MMS dose (150 mg/kg), we observe cell death in the cerebellar granule cells, the cell type which comprises 99% of the granular layer of the cerebellum. Following MMS treatment, there is a striking change in cerebellar morphology in *AagTg* mice. We observe severe cerebellar lesions containing numerous pyknotic nuclei surrounded by white spaces, indicative of edema (Figure 4A). Pyknotic nuclei, but not edema, are also observed, albeit at a lower frequency, in the cerebella of treated WT mice, whereas the cerebella of treated *Aag^−/−^* mice are indistinguishable from untreated mice (Figure 4A). The regions of edema were quantitated using image analysis software; examples of colorized lesions are shown in the lowest panel of Figure 4A. In untreated mice, no edema is observed (Figure 4B). However, 24 h following MMS (150 mg/kg), there is an obvious increase in edema in *AagTg* mice compared to either WT or *Aag^−/−^* mice, and a trend towards an increase in WT compared to *Aag^−/−^* mice (p = 0.308), suggesting that *Aag^−/−^* mice are protected against MMS-mediated cerebellar toxicity. Here we illustrate that MMS treatment results in severe cerebellar damage that is Aag-dependent. Although cerebellar damage has been described following treatment of early postnatal mice (PND3) with the alkylating agents methylazoxymethanol and mechlorethamine, to our knowledge, it has not previously been demonstrated following treatment of adult mice [49], [50].

### Aag-dependent, MMS-induced cerebellar degeneration results in impaired motor function

**Figure 5:**
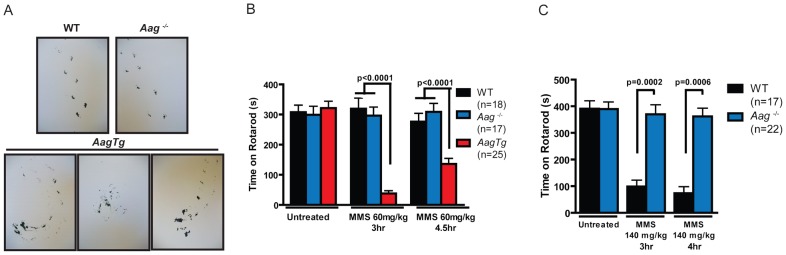
MMS induces an Aag-dependent decrease in motor function. (A) Representations of gait are shown for WT (n = 3), *Aag* ^−/−^ (n = 3) and *AagTg* (n = 3) mice three hours following MMS treatment (90 mg/kg). (B) Rotarod performance is shown for WT (n = 18), *Aag* ^−/−^ (n = 17) and *AagTg* (n = 25) mice under untreated conditions and following MMS treatment (60 mg/kg). Data represent mean ± SEM. (C) Performance for the rotarod challenge is shown for WT (n = 17) and *Aag* ^−/−^ (n = 22), 3 and 4 h following MMS treatment (140 mg/kg). Data represent mean ± SEM. All the mice in this figure are on a pure C57BL/6 background.

Given the importance of the cerebellum in coordinating motor function, we investigated whether MMS-induced cerebellar lesions result in diminished motor control. Decreased mobility was observed in all genotypes following a high MMS dose (150 mg/kg) (data not shown), so we reduced the MMS dose (90 mg/kg) for gait comparisons between WT, *Aag^−/−^*, and *AagTg* mice. The gait of all genotypes was indistinguishable under untreated conditions (Figure S4). However, three hours following MMS treatment, the gait of WT and *Aag^−/−^* mice is unchanged whereas *AagTg* mice exhibit severe gait abnormalities including immobility, circling, and walking backwards (Figure 5A). We quantitated motor defects by performing an accelerating speed rotarod test. To ensure all genotypes were capable of performing for >30 seconds on the rotarod test, we further reduced the MMS dose (60 mg/kg). Without MMS exposure, all genotypes performed comparably (Figure 5B). However, three hours following MMS treatment, we observed a dramatic decrease in rotarod performance for *AagTg* mice compared to WT and *Aag^−/−^* mice (Figure 5B). Although the *AagTg* mice slightly improved their performance by 4 hours, it remained significantly decreased compared to WT and *Aag* ^−/−^ mice (Figure 5B). Treated WT and *Aag^−/−^* mice performed similarly to untreated mice, indicating that MMS at this dose (60 mg/kg) caused no motor dysfunction in WT or *Aag^−/−^* mice.

We next increased the MMS dose to one that resulted in obviously impaired motor function in WT mice (140 mg/kg), and again examined motor function using the rotarod test (Figure 5C); the *AagTg* mice could not be included in this experiment due to their extreme sensitivity at this MMS dose. Strikingly, even at this high dose, *Aag^−/−^* mice remain completely protected against MMS-induced motor dysfunction; they not only exhibit significantly better performance than WT mice, but their performance following MMS remains the same as in untreated conditions (Figure 5C). This observation is consistent with the absence of histological cerebellar lesions in *Aag^−/−^* mice following MMS (150 mg/kg) treatment (Figure 4A). Together, these data underscore the importance of BER coordination in neuronal homeostasis; MMS induces cerebellar degeneration that is exacerbated by imbalanced BER in *AagTg* mice. Perhaps most importantly, the elimination of BER initiation in *Aag^−/−^* mice completely suppresses MMS-induced cerebellar toxicity.

### The absence of Parp1 suppresses Aag-dependent MMS-induced toxicity in several tissues

**Figure 6:**
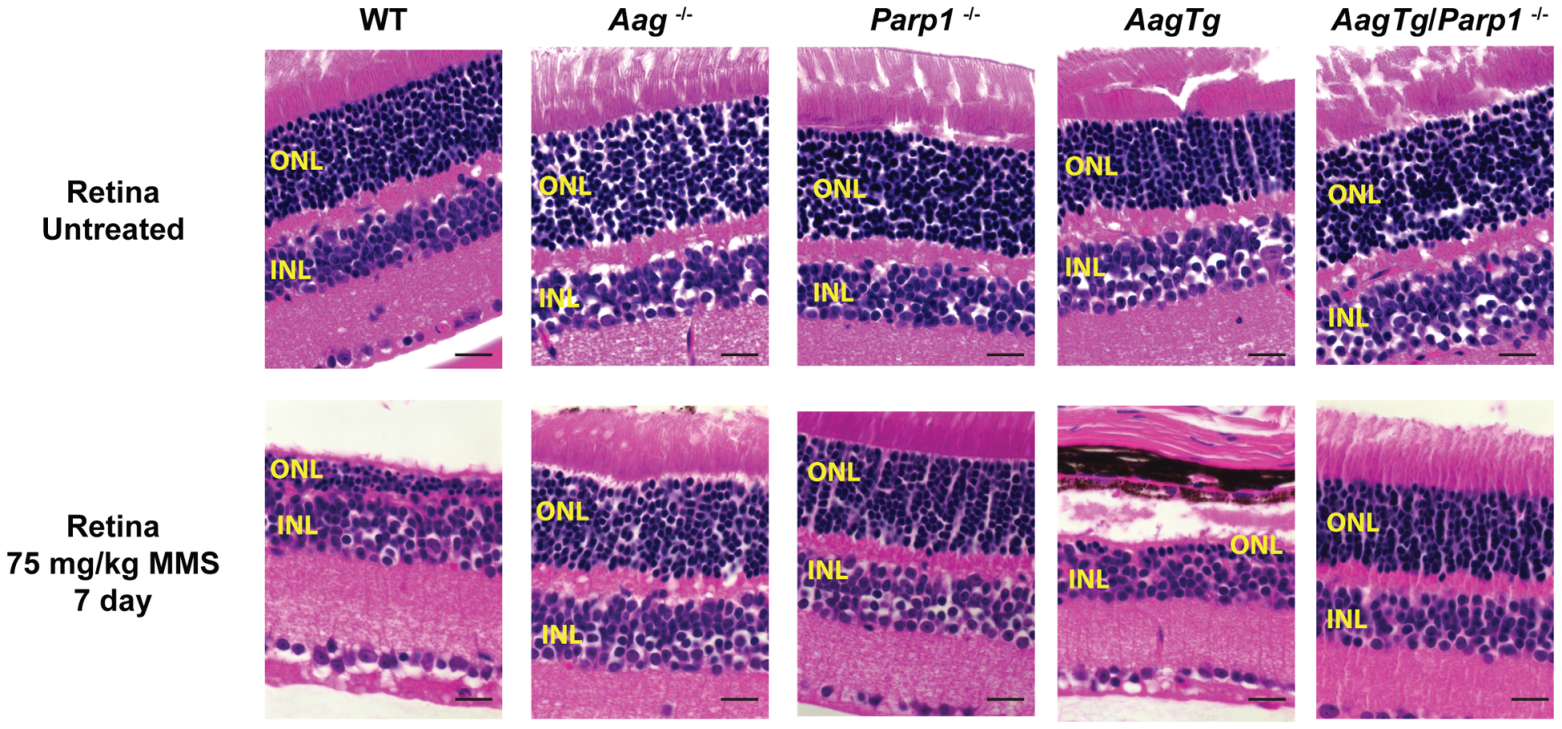
Parp1 deficiency protects against Aag-dependent, MMS-induced toxicity in retina photoreceptors. H&E stained retinal sections for WT, *Aag* ^−/−^, *Parp1* ^−/−^, *AagTg*, and *AagTg*/*Parp1* ^−/−^ under untreated conditions or 7 d following MMS treatment (75 mg/kg). Scale bar is 15 µm. Representative images for n = 5 mice/genotype are shown. All the mice used in this figure are mixed C57BL/6:129S background. ONL, Outer nuclear layer; INL, inner nuclear layer.

Given the role for Parp1 in mediating alkylation toxicity, we next set out to determine whether eliminating Parp1 could modulate the MMS-induced cytotoxicity observed in WT and *AagTg* mice; to explore this possibility, we utilized *Parp1* ^−/−^ mice [51]. We first investigated MMS-induced retinal degeneration (RD). As previously illustrated, MMS induces the selective degeneration of the photoreceptors in the retinal outer nuclear layer (ONL) in WT mice, whereas juxtaposed retinal layers are unaffected [29]. While *Aag* ^−/−^ mice are completely protected from such RD, *AagTg* mice are hypersensitive compared to WT and *Aag* ^−/−^ mice, as observed by decreased number of cells found within the ONL (Figure 6 and [29]). Strikingly, we observe that *Parp1* ^−/−^ mice are also completely protected against MMS-induced RD (Figure 6). Moreover, MMS-induced RD is completely abrogated in *AagTg/Parp1* ^−/−^ mice, indicating that Parp1 deficiency completely suppresses the Aag-dependent MMS-hypersensitivity in photoreceptors of *AagTg* mice (Figure 6). To confirm that Parp1 enzymatic activity is stimulated during MMS-induced Aag-dependent RD, we evaluated Parp1 activation by immunodetection of the PAR polymer. Figure S5 shows that increased PAR polymer staining is observed 24 h following MMS treatment, in an Aag-dependent manner, confirming that MMS-induced RD is preceded by Parp1 activation. Similarly, we observe that *Parp1* deficiency is able to suppress the pancreatic β-cell death observed in *AagTg* mice following an acute MMS treatment (Figure S6). Together, these data indicate that the BER intermediates exert their toxicity through the hyperactivation of Parp1. Further, we find that deletion of Parp1 prevents MMS-induced toxicity and that both Aag and Parp1 are required for MMS-induced cell death.

**Figure 7:**
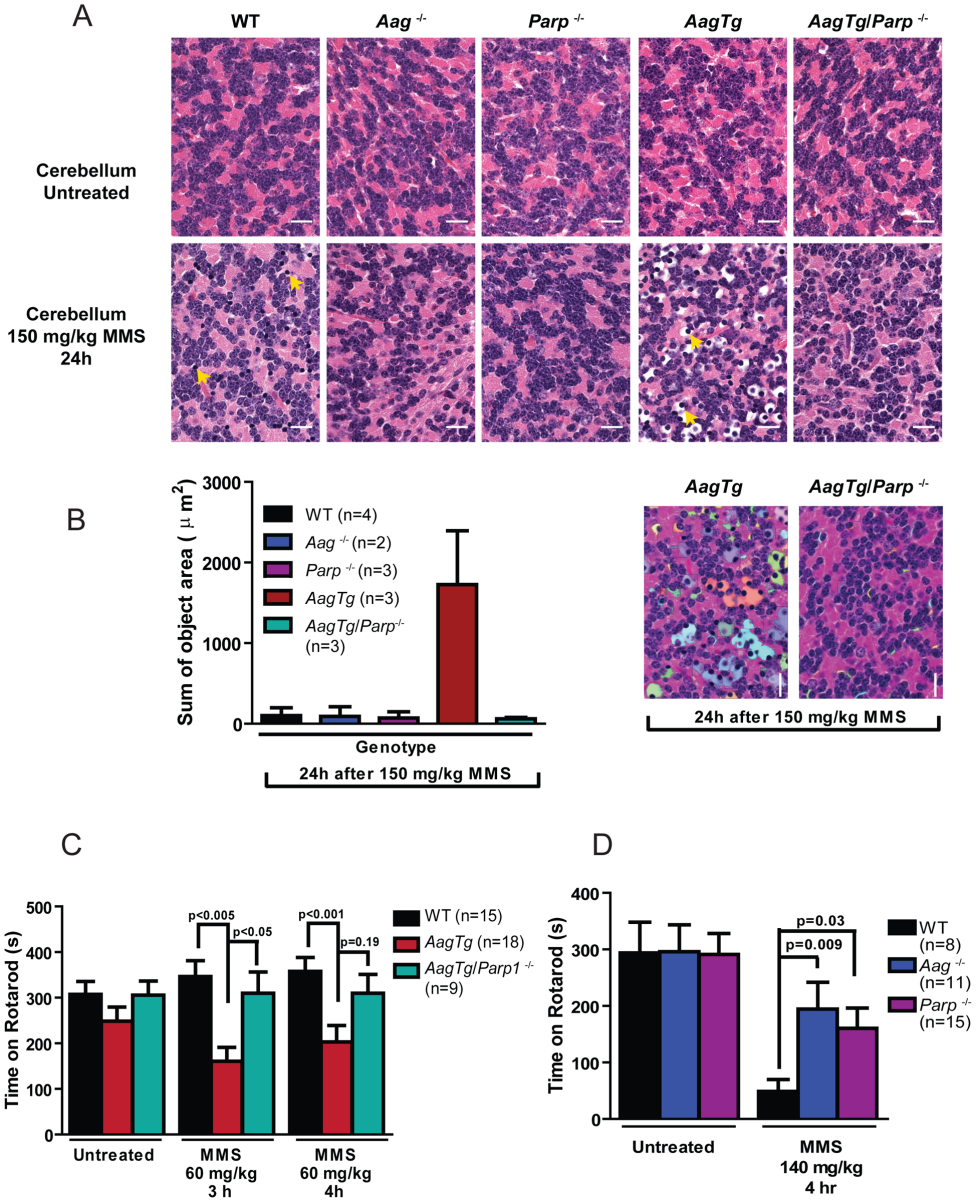
Parp1 deficiency protects against Aag-dependent, MMS-induced motor dysfunction. (A) H&E stained cerebellar sections are shown from WT, *Aag* ^−/−^, *Parp1* ^−/−^, *AagTg*, and *AagTg*/*Parp1* ^−/−^ under untreated conditions or 24 h following MMS treatment (150 mg/kg). Scale bar is 15 µm. Representative images for n = 5 mice/genotype are shown. (B) Quantitation of cerebellar phenotype is shown. Three or more images/cerebella were quantitated per mouse, and 3 mice per genotype analyzed for quantitation; the average sum of object area (edema) per image is presented. (C) Performance during the rotarod challenge in WT (n = 15), *AagTg* (n = 18), and *AagTg*/*Parp1* ^−/−^ (n = 9) is illustrated under untreated conditions and following MMS treatment (60 mg/kg). (D) Performance for the rotarod challenge is shown for WT (n = 8), *Aag* ^−/−^ (n = 11), and *Parp1* ^−/−^ (n = 15) mice four hours following MMS treatment (140 mg/kg). All the mice used in this figure are mixed C57BL/6:129S background. All data represent mean ± SEM.

We next investigated the requirement for Parp1 in MMS-induced cerebellar degeneration. While MMS (150 mg/kg) induces severe cerebellar lesions in *AagTg* mice (Figure 4A), Parp1 deficiency completely suppresses this Aag-dependent, alkylation-induced cerebellar toxicity (Figure 7A). Image analysis confirms that the drastic increase in the edema in MMS-treated *AagTg* mice was completely abrogated in *Aag*Tg/*Parp1* ^−/−^ mice (Figure 7B). Consistent with rescue of cerebellar lesions in *AagTg/Parp1^−/−^* mice (Figure 7B), we illustrate using gait analysis that Parp1 deficiency prevents the motor dysfunction observed following MMS treatment in *AagTg* mice (Figure S7). Additionally, rotarod assays were performed in WT, *AagTg* and *AagTg/Parp^−/−^* mice to quantitate motor function. Following MMS (60 mg/kg), the motor function in WT mice remain unaffected, whereas *AagTg* mice exhibit significantly diminished performance (Figure 7C). Importantly, following MMS, *AagTg*/*Parp1* ^−/−^ mice perform just as well as WT mice, indicating that the Aag-dependent MMS-hypersensitivity in the cerebella of *AagTg* mice is completely dependent on Parp1 (Figure 7C). Further, the Parp1 deficiency was sufficient to prevent the motor dysfunction observed at high MMS doses. As described above, at high MMS (140 mg/kg), WT mice exhibit severe motor dysfunction (Figure 5C and Figure 7D); however we find that like *Aag* ^−/−^ mice, *Parp1* ^−/−^ mice are protected against MMS-induced motor dysfunction and exhibit enhanced rotarod performance compared to MMS-treated WT mice (Figure 7D). The mice in this experiment are on a mixed C57Bl/6:129S6 background, and the slight decrease in rotarod performance observed here, compared to Figure 5, can be attributed to differences in genetic background, as previously shown [52], [53]. Together, these data (Figure 6, Figure 7, and Figure S6) indicate that both Aag and Parp1 are required for the severe MMS-mediated cytotoxicity observed in retinal photoreceptors, pancreatic β-cells, and cerebellar granule cells.

## Discussion

Using *AagTg* mice, we have investigated the *in vivo* consequences of imbalanced BER. Although increased human AAG activity was recently linked to elevated lung cancer risk [7], [9] and decreased survival of glioma patients [54], [55], increased Aag activity in mice does not affect spontaneous tumorigenesis or overall longevity. However, dramatic *in vivo* consequences of imbalanced BER were revealed upon treating *AagTg* mice with alkylating agents. *AagTg* mice exhibited increased whole-animal lethality to both S_N_1 and S_N_2 methylating agents, but not to other genotoxic agents. Our data suggest that under basal conditions, the level of BER intermediates produced during the repair of spontaneous DNA damage is readily accommodated by the downstream BER enzymes. However, in the presence of higher levels of DNA base damage generated by alkylating agents, Aag initiates BER at a rate such that downstream BER enzymes may be unable to efficiently process the toxic BER intermediates, resulting in cell death and tissue damage, and potentially in the death of the animal (Figure 1). Therefore, it may be predicted that patients exhibiting increased AAG activity may exhibit increased sensitivity or more detrimental side-effects to alkylating chemotherapeutic agents. Indeed, AAG expression does predict temozolomide sensitivity in glioblastoma and ovarian cancer cell lines [48], [56], and AAG expression inversely correlates with survival of glioma patients following treatment [54]. Together, the data presented here as well as published findings provide justification for an epidemiological study examining alkylation sensitivity in correlation with AAG activity levels.

Imbalanced BER in *AagTg* mice confers increased MMS sensitivity to cells in the thymus, spleen, bone marrow, retina, pancreas, and cerebellum. Further, Aag activity predicts MMS toxicity *in vivo*, such that relative sensitivities are as follows: *AagTg*>WT>*Aag* ^−/−^. However, Aag activity is not the sole determinant of MMS-mediated tissue cytotoxicity; numerous tissues in the *AagTg* mice exhibit ∼5–8 fold increases in Aag activity (e.g. heart, kidney, liver) but show no evidence of increased MMS sensitivity (Figure S2 and data not shown), underscoring the importance of cellular context in determining Aag-mediated alkylation sensitivity. It remains to be determined why only subsets of cell types are susceptible to Aag-mediated alkylation toxicity. In the highly-proliferative thymus, spleen, and bone marrow, unrepaired BER intermediates presumably result in replication fork collapse and DSBs, thus triggering cell death. However, pancreatic β-cells are not highly-proliferative and both the adult cerebellar granule cells and retinal photoreceptor cells are non-replicating, post-mitotic tissues [57], indicating cell death must be replication-independent. It is important to note that even within tissues exhibiting MMS sensitivity, toxicity is not uniform across all cell types in the tissue. For example, only the ONL of the retina undergoes MMS-induced degeneration whereas the juxtaposed retinal layers remain intact. Similarly, following MMS treatment, cerebellar purkinje cells remain unaffected while neighboring cerebellar granule cells are ablated. Why are some cells so sensitive, whereas others are resistant? The activity of downstream BER proteins in the sensitive cells may simply be insufficient to process accumulating BER intermediates; this and other possibilities are currently under investigation. Together, our data emphasize the concept that BER imbalance and the resulting intermediates can profoundly affect cellular and tissue homeostasis, and reveal that in certain contexts, the absence of DNA repair can actually be beneficial to an organism.

Although it has been known for 30 years that Parp inhibition potentiates alkylation-induced toxicity [58], the recent discovery of synthetic lethality in BRCA1/2 homologous recombination-deficient tumors upon Parp inhibition has renewed intense interest in using PARP inhibitors for cancer chemotherapy [59], [60]. However, in stark contrast to this well-documented potentiation of alkylation toxicity by Parp inhibitors [30], [61], we observe complete suppression of alkylation toxicity by genetic deletion of Parp1. This is likely due to multiple inherent differences between Parp1 deficiency and Parp inhibition [62]. The main difference is the proposed trapping of Parp1 on DNA substrates by Parp inhibitors, which thereby prevents BER and interferes with replication [63], [64]. Although this ‘DNA trapping’ phenomenon has been demonstrated for many Parp inhibitors, it may not be the case for all Parp inhibitors [64]–[67]. In fact, uncovering the relationship between the inhibition of the catalytic activity of Parp, the potency of DNA trapping, and overall toxicity by Parp inhibitors has recently garnered interest [66]. Another difference between Parp1 deficiency and Parp inhibition resides in the fact that Parp inhibitors are not specific for the inhibition of Parp1, but can potentially inhibit the catalytic activity of 17 other members of the Parp superfamily [68]. Regardless of the differences between Parp1 deletion and Parp inhibition, suppression of alkylation toxicity upon treatment of cultured cells with Parp inhibitors is not unprecedented [43], [69]–[71].

Although Parp1 deficiency mechanistically differs from Parp inhibition, it was surprising to observe that Parp1 deficiency was capable of completely suppressing MMS hypersensitivity in numerous tissues under conditions of imbalanced BER (*AagTg* mice). This *in vivo* data reveals that Parp1 acts downstream of Aag to govern alkylation sensitivity, presumably through Parp1's alternate function in mediating programmed necrosis (Figure 1B) [12], [71]. Interestingly, interrupting Parp1's function has been shown to be protective in several other models of neuronal damage including ischemia/reperfusion and glutamate excitotoxity [72]–[74], and retinal degeneration induced by ischemia/reperfusion or by treatment with PDE6 inhibitor, which mimics the *rd1* mutation [75], [76]. Whether Aag also plays a role upstream from Parp1 in these modes of tissue damage remains to be determined.

Using genetic experiments, we show here that modestly increased Aag activity results in dramatic increases in tissue and whole-animal sensitivity to alkylating agents. Given that human AAG activity varies greatly among healthy individuals (Figure 2), and also that BER protein levels are known to be altered in numerous human cancers [77]–[79], the resulting imbalanced BER may have dramatic consequences in patients undergoing chemotherapy involving alkylating agents [6]. Moreover, PARP1 expression and activity varies greatly in human tumors [80], and SNPs in *PARP1* have been associated with numerous cancers [81]–[83]. We illustrate that Parp1 deficiency protects against alkylation sensitivity at both the tissue and whole-animal level. Therefore, decreased PARP1 activity may result in a decreased response during a chemotherapeutic regimen. Indeed, leukemic patients expressing decreased PARP1 levels exhibit resistance to standard chemotherapy therapy [84], [85]. Taken together, our findings illustrate that monitoring for both BER imbalance and PARP1 expression is warranted prior to selecting a chemotherapeutic regimen that includes alkylating agents.

## Materials and Methods

### Ethics statement

The MIT Committee on the Use of Humans as Experimental Subjects reviewed and approved the research involving human subjects. Written informed consent was obtained from all participants. All animal procedures were approved by the MIT Committee on Animal Care.

### Animals

*Aag^−/−^* and *Aag* transgenic (*AagTg*) mice were described previously [21], [29]. *Parp ^−/−^* mice (Jackson Laboratories) were described previously [51]. Mice were fed a standard diet *ad libitum*, housed in an AAALAC-accredited facility, and euthanized by CO_2_ asphyxiation. Additional details about the mice utilized are included in the Methods S1.

### Reagents

Methyl methanesulfonate (MMS), chloroacetaldehyde (CAA), N-methyl-N-nitrosurea (MNU), and Mitomycin C (MMC) were obtained from Sigma (St. Louis, MO). Azoxymethane (AOM) was obtained from the Midwest Research Institute, NCI Chemical Repository.

### Treatments

Approximate LD_50_ (whole-animal sensitivity to genotoxic agents) was determined as in Deichmann and LeBlanc [86]. Acute MMS treatments were performed by i.p. injecting mice with varying doses of MMS (60–150 mg/kg).

### Tissue processing and histopathology

Tissues were processed at the David H. Koch Institute for Integrative Cancer Research, Histology Core Facility; they were paraffin-embedded, sectioned at 5 µm, and stained with hematoxylin and eosin (H&E). All H&E stained slides were blindly analyzed by a pathologist (R.T.B) for the cause of death as well as for the identification of any tumors/lesions.

### Image quantitation

Volocity (Perkin Elmer) image analysis software was used to quantitate edema, as observed by white spaces in cerebellar histological sections. Thresholding was performed using the Red/Green/Blue quantitation tool and objects smaller than 5 microns were excluded. The sum of all object areas/image was calculated and greater than 3 representative images were analyzed for each of 3 animals.

### *Ex vivo* bone marrow clonogenic assays

Bone marrow cells were harvested from the femurs of 6–12 weeks old WT, *Aag* ^−/−^ and *AagTg* mice. Cells were treated with varying dose of MMS, washed, resuspended in complete media mixed with methylcellulose (Stem Cell Technologies), plated in duplicate and the percent survival calculated as in [28]. Experiments were performed in triplicate.

### Evaluation of Aag activity in mouse tissues

Cell extracts were made from tissues harvested from WT, *AagTg*, and *Aag^−/−^* mice. Tissues were sonicated in Aag glycosylase assay buffer (20 mM Tris-Cl pH 7.6, 100 mM KCl, 5 mM EDTA, 1 mM EGTA and 5 mM β-mercaptoethanol) with protease inhibitors. Protein concentration was measured using micro BCA Kit (Pierce). Glycosylase assays were performed as previously published [21]. A [^32^P]γ-labeled double-stranded 25mer oligonucleotide containing a single centrally located hypoxanthine residue [5′-GCAATCTAGCTTTTT(Hx)CGATGTATGC-3′] was incubated with an amount of extract determined to be in the linear range for activity at 37°C for 1 h. The resulting AP sites were cleaved by incubation with 0.1 N NaOH at 70 °C for 20 min. A phosphorimager was used to visualize and quantitate Aag DNA glycosylase activity.

### Evaluation of AAG activity in PBMC

Peripheral blood samples were obtained from healthy individual at the MIT Catalyst Clinical Research Center, and PBMC were isolated using standard Ficoll-Paque (Sigma) density gradient centrifugations. The glycosylase assay was performed as above with the following exceptions; the [^32^P]γ-labeled lesion-containing oligonucleotide used was 5′-GCAATCTAGCCA(εA)GTCGATGTATGC-3′, and the glycosylase reaction was incubated for 37°C for 2 h.

### Evaluation of motor function

To capture gait abnormalities, the mouse hind paws were dipped into non-toxic paint; mice were placed in a closed container on a sheet of white paper and allowed to walk freely. The Ugo Basile 7650 Accelerating Rotarod was used to evaluate fore-limb and hind-limb motor coordination. The rotarod testing protocol was two weeks long; the first week examined motor function under untreated conditions and the second week following MMS-treatment. An accelerating speed rotarod protocol was used, which linearly accelerates the rotating rod from 4 to 40 RPM in 10 minutes. During each week, day 1 to 4 consisted of training the mice for two 5-minute runs to familiarize the mice with the apparatus. On day 5, the mice underwent two tests, with an hour rest between the tests; the time spent on the rotatod is recorded as a measurement of performance. The 2^nd^ week follows the same schedule, 4 days training and test on the fifth day. However, on the 5^th^ day of the second week, the mice were treated with either 60 or 140 mg/kg MMS by i.p. injection and the test performed 3 and 4–4.5 hours post-treatment.

### Immunofluorescence

5 µm unstained sections were deparaffinized and rehydrated in a graded ethanol series, incubated in citrate buffer and thermally processed for epitope retrieval. Sections were permeabilized three times (5 minutes) with PBS-T (1× PBS+0.1% Triton X-100). Non-specific antibody binding sites were blocked by incubating sections with 1× PBS-T+heat-inactivated goat serum (HIGS; 10%) for 30 minutes. Sections were then incubated with primary anti-PAR antibody (1∶250; BD Pharmingen) for 2 hours at RT. After 5 washes in PBS-T, sections were incubated for 30 min with secondary antibody DyLight 488 (1∶1000; Vector Labs). Nuclear counterstaining was done using TOPRO-3 (Invitrogen). All staining was performed in humidified chambers. A Zeiss Axiovert LSM 510 META confocal microscope (Germany) with a 63× oil objective was used to image the retinal sections. Images were viewed and analyzed using LSM Image Browser.

### Statistics

Statistical analyses were performed using GraphPad Prism software. Data are presented as mean ± SEM or mean ± SD (as stated in figure legends). Statistical significance was determined using unpaired t-test or two-way ANOVA. Kaplan-Meier survival curves were generated and survival differences determined using the Log-Rank test. A p-value is considered significant if less than 0.05.

## Supporting Information

Figure S1 Evaluation of *AagTg* mice. (A) Aag activity is illustrated for a panel of tissues in WT, *Aag* ^−/−^ and *AagTg* mice. *In vitro* glycosylase assays were performed on tissues isolated from n = 3 animals. (B) Kaplan Meier Survival curves are shown for an aging cohort of WT (n = 19), *Aag* ^−/−^ (n = 27) and *AagTg* (n = 26) mice.
(TIF)

Figure S2 Aag transgene expression does not result in MMS atrophy in all tissues. Tissue weights of the heart, left kidney, brain, left gonadal fat pad, and left gastrocnemius/soleus skeletal muscles were taken in untreated and 24 h post MMS treatment (75 mg/kg). The mice utilized in this experiment were age-matched males on a pure C57Bl/6 background.
(TIF)

Figure S3 MMS induces pancreatic β-cell death *AagTg* mice. H&E stained slides of pancreatic β-islets (outlined in yellow) from WT, *Aag* ^−/−^ and *AagTg* mice either in untreated conditions or 24 h following MMS treatment (150 mg/kg). Untreated sections show healthy pancreatic histology. Following MMS treatment, only *AagTg* exhibit evidence of toxicity within the β-cells, as illustrated by pyknotic and fragmented nuclei (shown by yellow arrows). Very few intact nuclei are observed in the pancreatic β-islet of the MMS-treated *AagTg* mice (green arrows). Representative images are shown of n>3 experiments. Scale bar is 12 µm.
(TIF)

Figure S4 Untreated mice exhibit similar gait. Representations of gait are shown for WT (n = 3), *Aag* ^−/−^ (n = 3), and *AagTg* (n = 3) mice prior to MMS treatment.
(TIF)

Figure S5 Aag-dependent Parp1 activation is observed in the retinal outer nuclear layer (ONL) following MMS treatment. Immunofluorescence staining with α-PAR antibody and TOPRO nuclear counterstain was performed on retinal sections from WT, *Aag* ^−/−^ and *AagTg* mice 24 h following MMS (75 mg/kg) treatment. ONL, outer nuclear layer; INL, inner nuclear layer.
(TIF)

Figure S6 Parp1 deficiency protects against alkylation-induced pancreatic β-cell death *AagTg* mice. H&E stained slides of pancreatic β-islets from WT, *Aag* ^−/−^, *Parp1* ^−/−^, *AagTg*, and *AagTg/Parp1* ^−/−^ mice either in untreated conditions or 24 h following MMS treatment (150 mg/kg). Representative images are shown of n>2 experiments. The pancreatic β-islets are centered in image and surrounded by pancreatic acinar cells. Untreated sections show healthy pancreatic histology. Following MMS treatment, only *AagTg* exhibit evidence of pancreatic β-cell toxicity, as illustrated by pyknotic and fragmented nuclei (yellow arrow). Very few intact nuclei are observed in the pancreatic β-islet of the MMS-treated *AagTg* mice (green arrows). Magnification is 60×; scale bar is 16 µm.
(TIF)

Figure S7 Parp1 deficiency protects against alkylation-induced gait abnormalities. Representations of gait are shown for WT (n = 3), *Parp1* ^−/−^ (n = 2), *AagTg* (n = 3) and *AagTg/Parp1* ^−/−^ (n = 2) mice shown three hours following MMS treatment (90 mg/kg).
(TIF)

Methods S1 Supplemental Materials and Methods.
(DOCX)

Table S1 Aag activity in Aag transgenic founder lines.
(DOCX)
