# Supplementary figures and images for: Aag DNA Glycosylase Promotes Alkylation-Induced Tissue Damage Mediated by Parp1
Source: PLoS Genet. 2013 Apr 4;9(4):e1003413. doi: 10.1371/journal.pgen.1003413 (PMC3617098; doi:10.1371/journal.pgen.1003413)

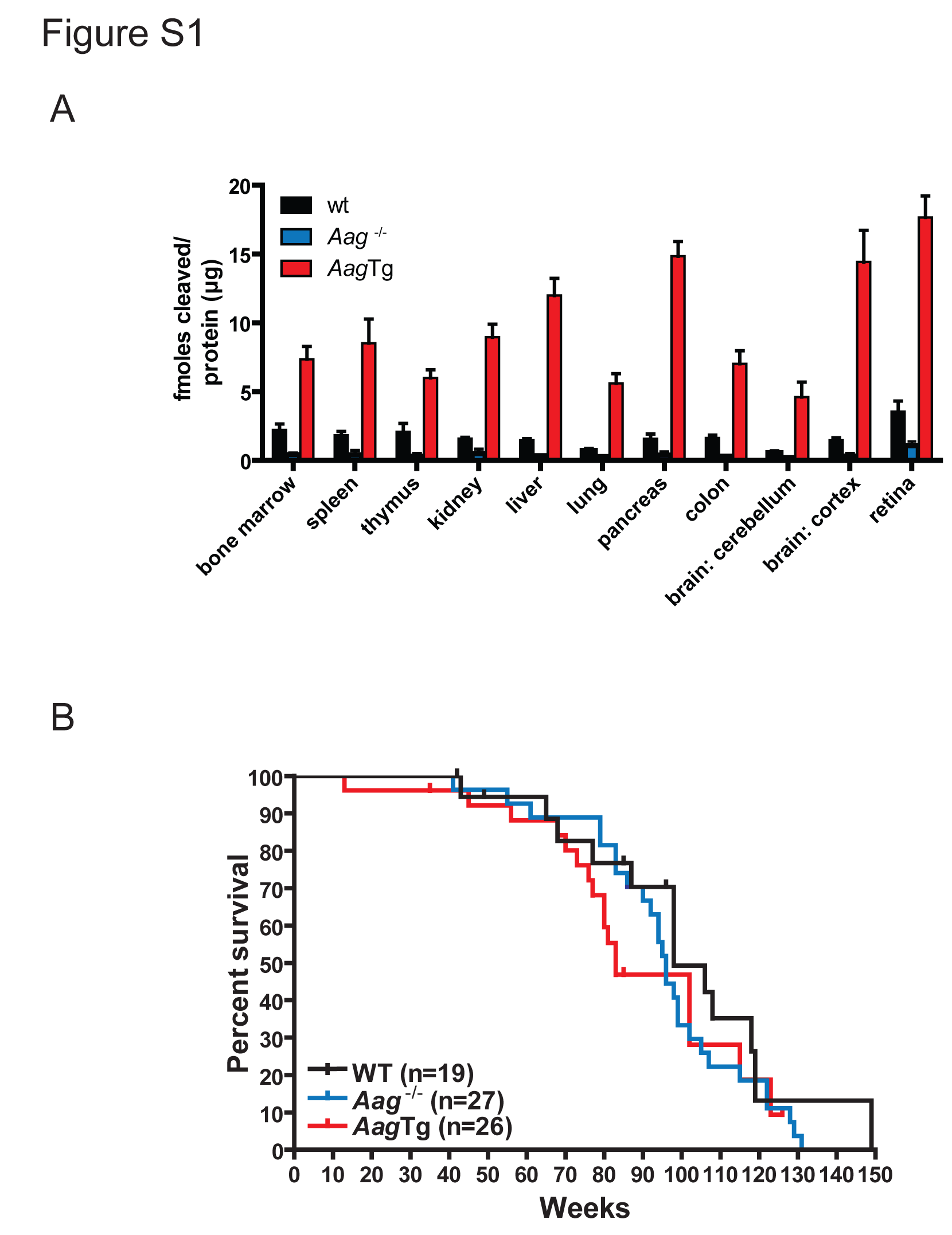

Supplement: Figure S1 — Evaluation of AagTg mice. (A) Aag activity is illustrated for a panel of tissues in WT, Aag −/− and AagTg mice. In vitro glycosylase assays were performed on tissues isolated from n = 3 animals. (B) Kaplan Meier Survival curves are shown for an aging cohort of WT (n = 19), Aag −/− (n = 27) and AagTg (n = 26) mice. (TIF) [file pgen.1003413.s001.tif]

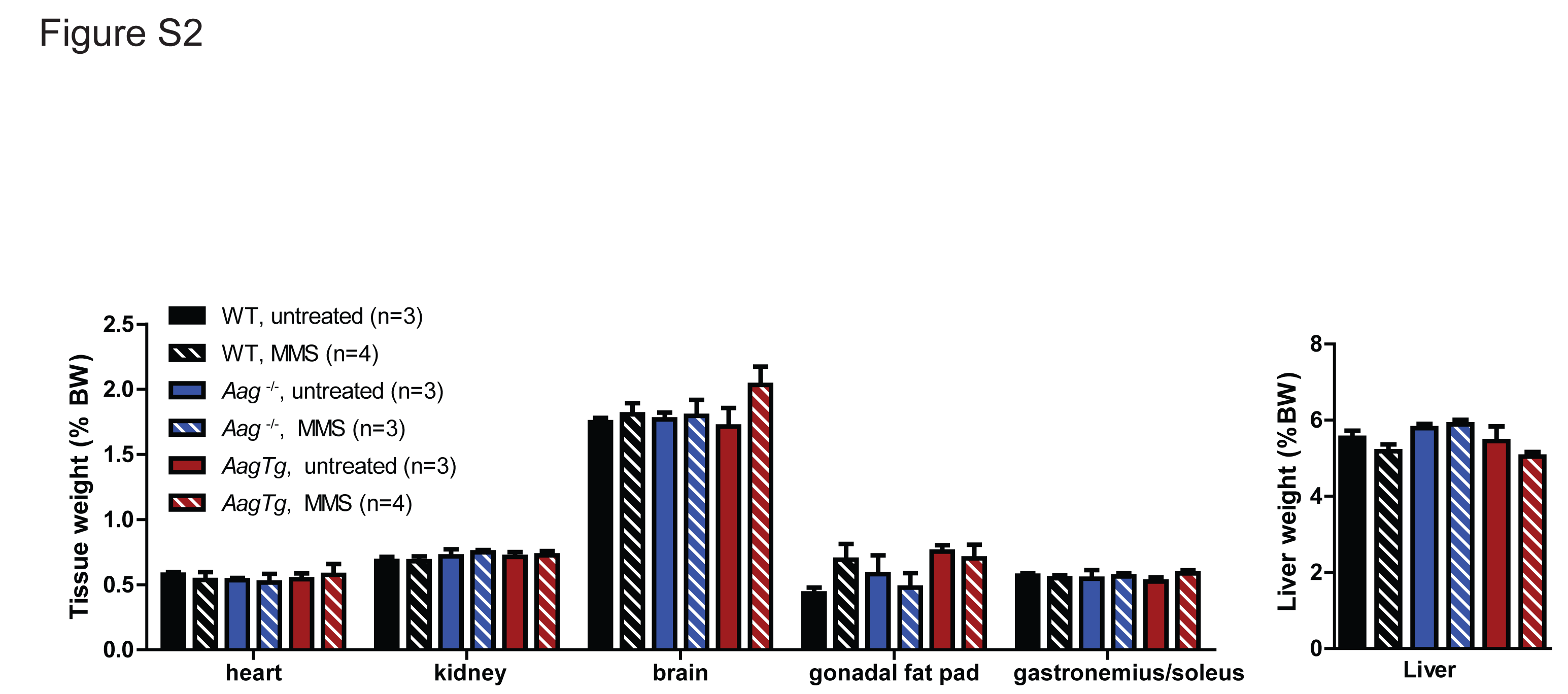

Supplement: Figure S2 — Aag transgene expression does not result in MMS atrophy in all tissues. Tissue weights of the heart, left kidney, brain, left gonadal fat pad, and left gastrocnemius/soleus skeletal muscles were taken in untreated and 24 h post MMS treatment (75 mg/kg). The mice utilized in this experiment were age-matched males on a pure C57Bl/6 background. (TIF) [file pgen.1003413.s002.tif]

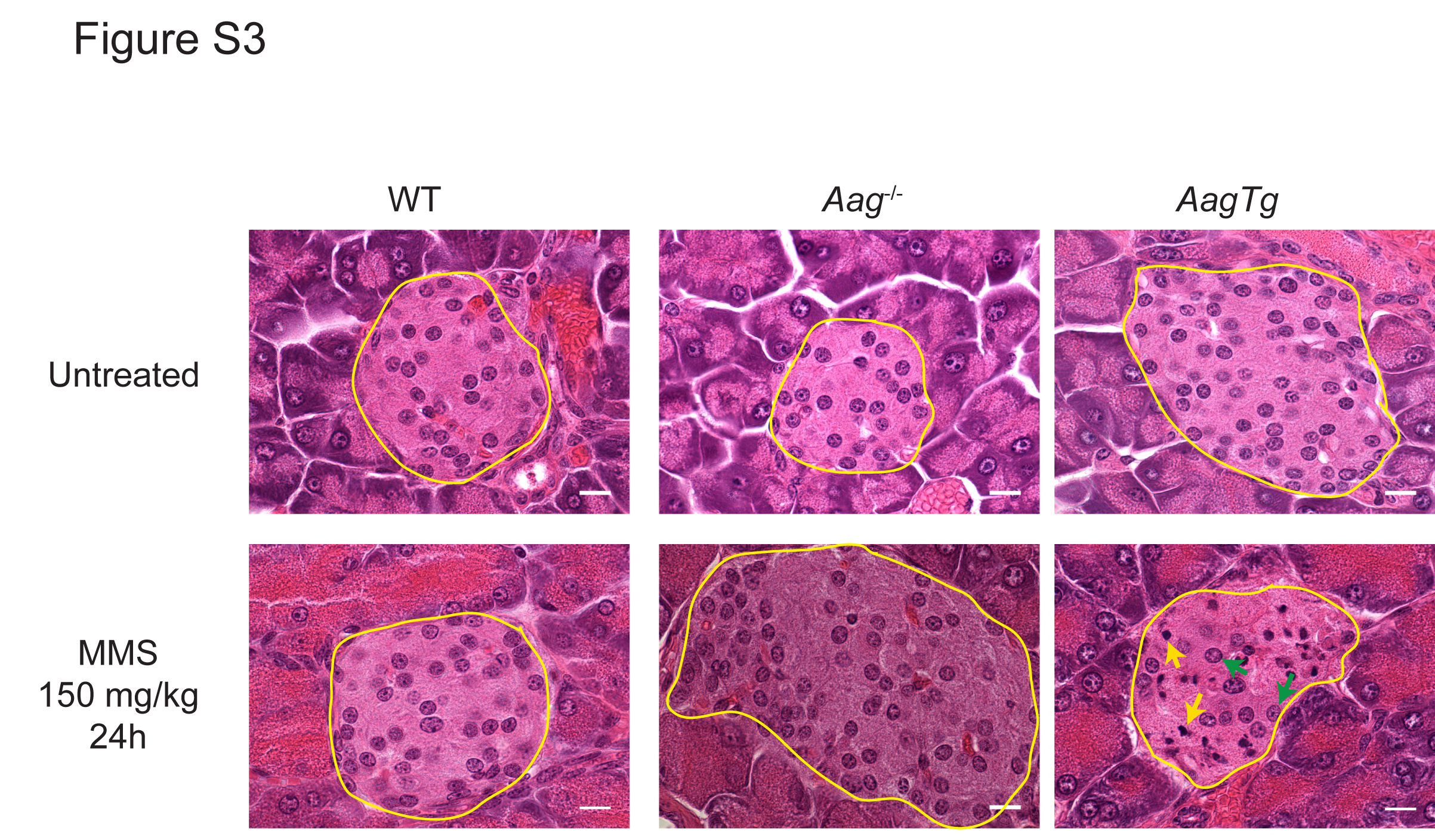

Supplement: Figure S3 — MMS induces pancreatic β-cell death AagTg mice. H&E stained slides of pancreatic β-islets (outlined in yellow) from WT, Aag −/− and AagTg mice either in untreated conditions or 24 h following MMS treatment (150 mg/kg). Untreated sections show healthy pancreatic histology. Following MMS treatment, only AagTg exhibit evidence of toxicity within the β-cells, as illustrated by pyknotic and fragmented nuclei (shown by yellow arrows). Very few intact nuclei are observed in the pancreatic β-islet of the MMS-treated AagTg mice (green arrows). Representative images are shown of n>3 experiments. Scale bar is 12 µm. (TIF) [file pgen.1003413.s003.tif]

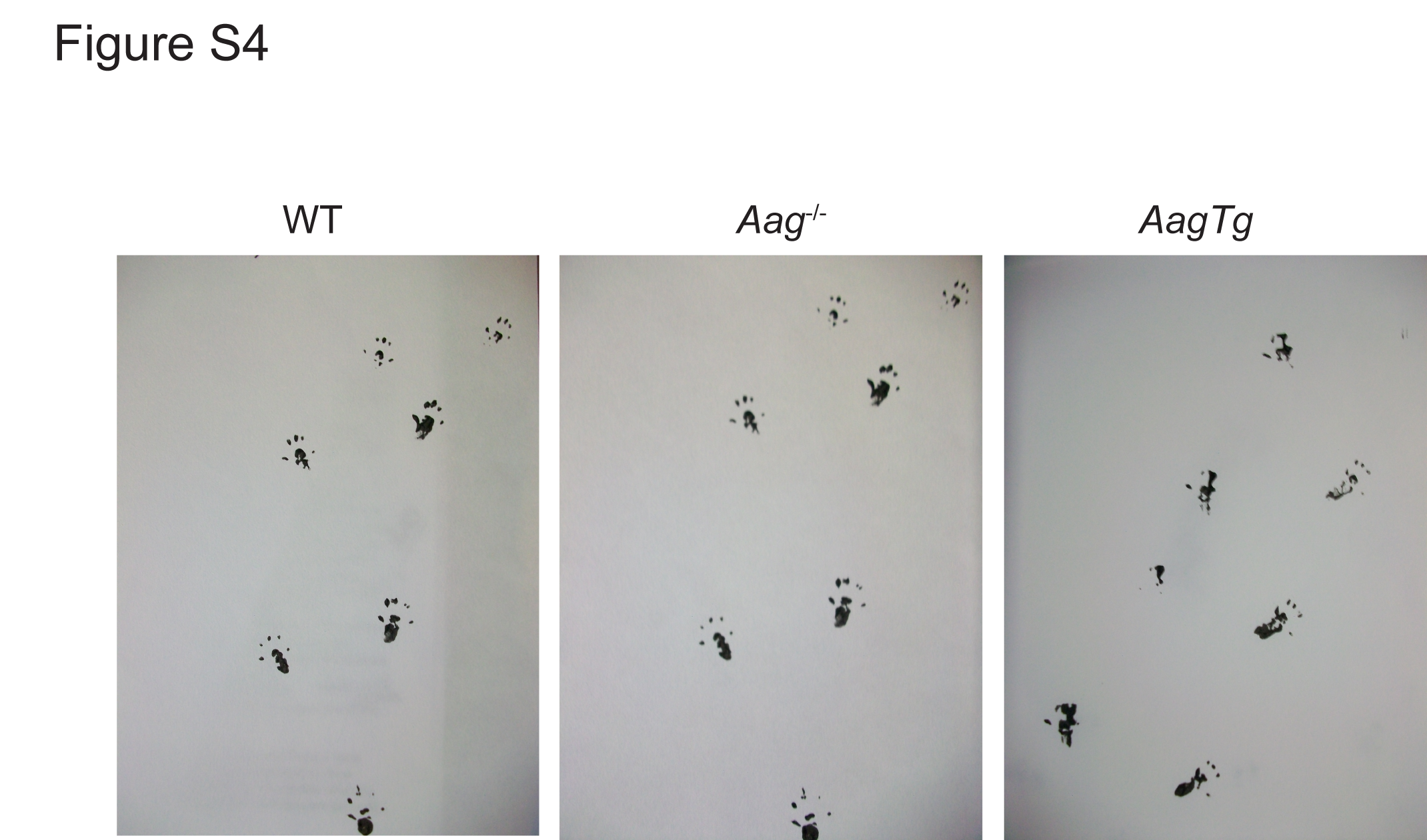

Supplement: Figure S4 — Untreated mice exhibit similar gait. Representations of gait are shown for WT (n = 3), Aag −/− (n = 3), and AagTg (n = 3) mice prior to MMS treatment. (TIF) [file pgen.1003413.s004.tif]

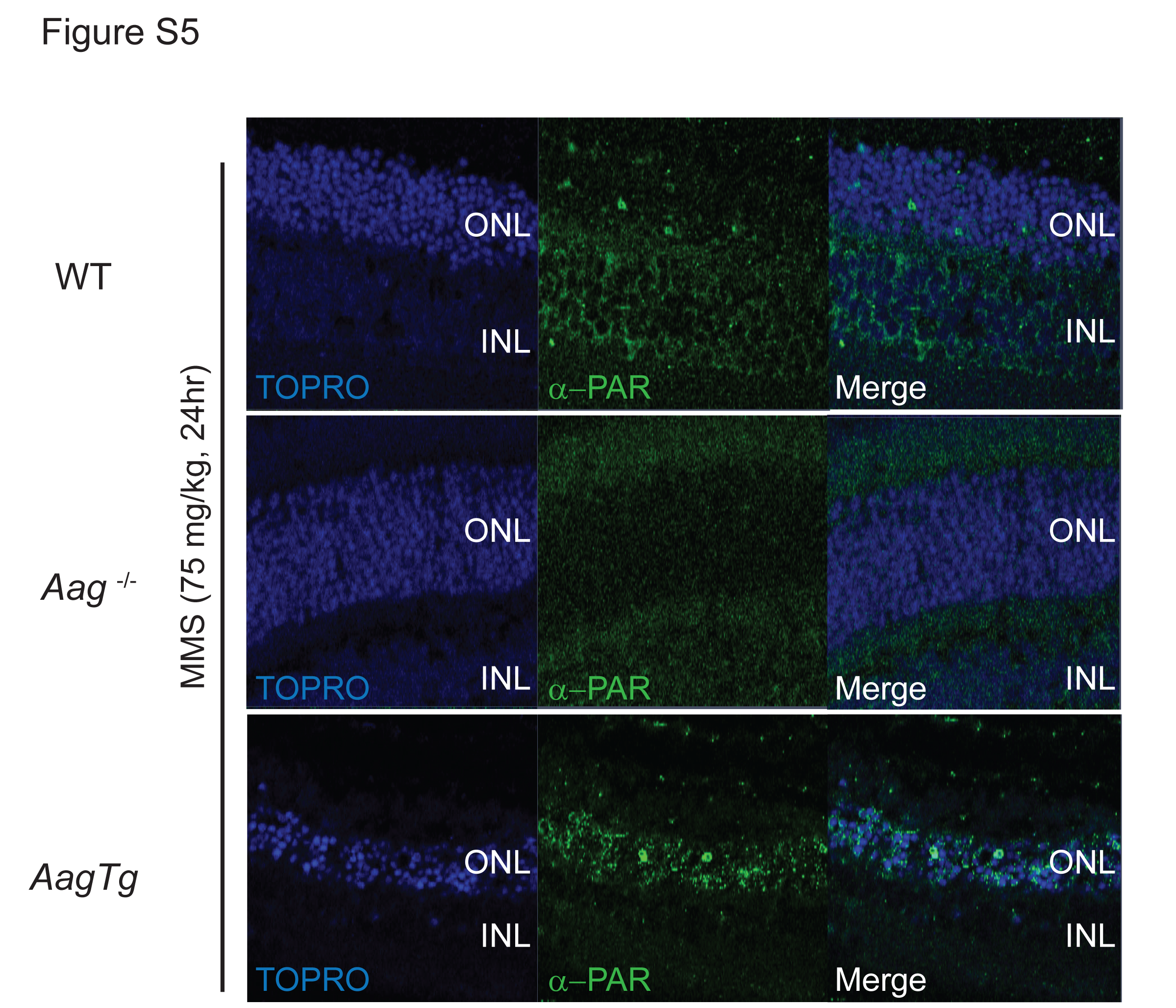

Supplement: Figure S5 — Aag-dependent Parp1 activation is observed in the retinal outer nuclear layer (ONL) following MMS treatment. Immunofluorescence staining with α-PAR antibody and TOPRO nuclear counterstain was performed on retinal sections from WT, Aag −/− and AagTg mice 24 h following MMS (75 mg/kg) treatment. ONL, outer nuclear layer; INL, inner nuclear layer. (TIF) [file pgen.1003413.s005.tif]

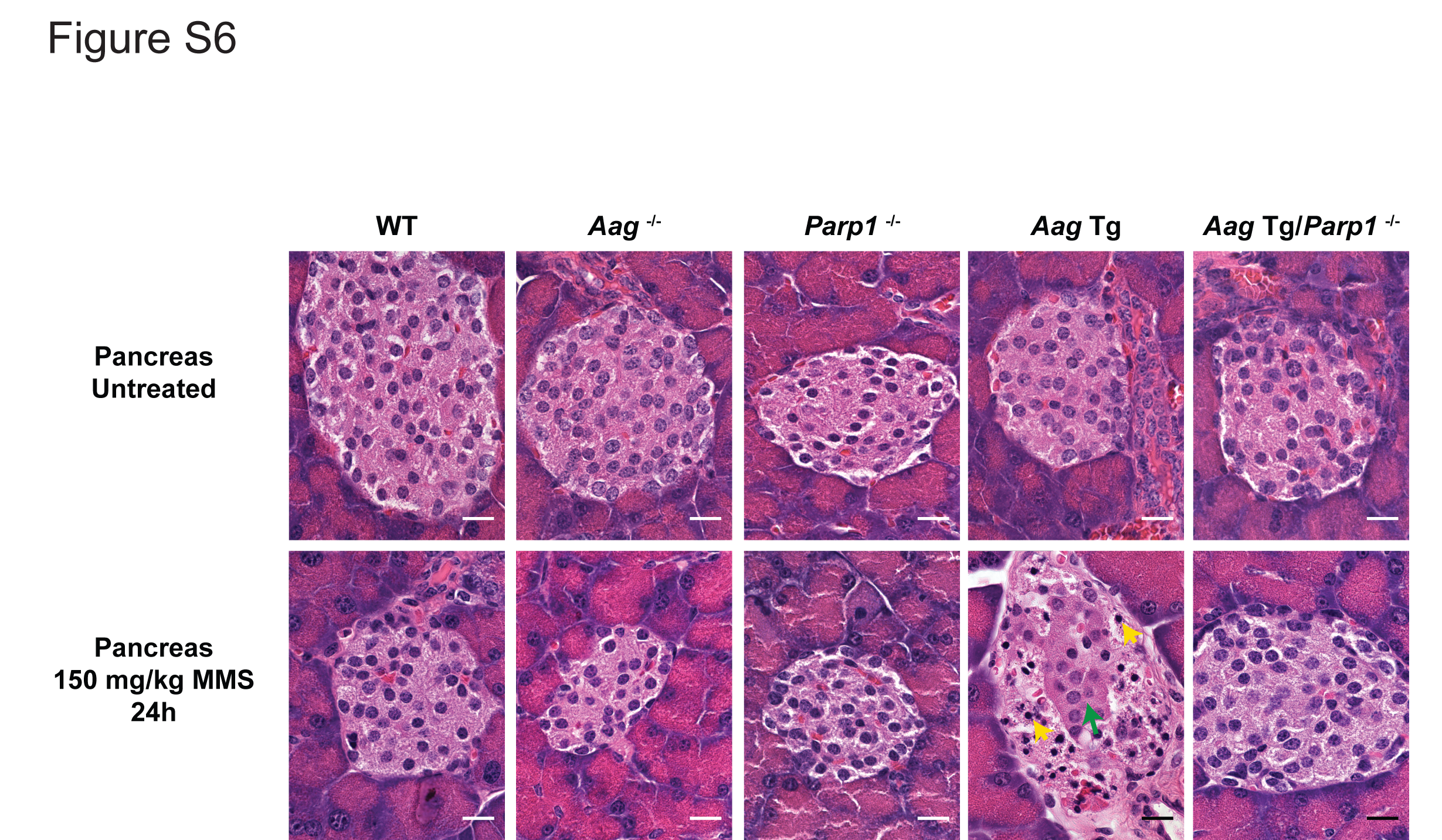

Supplement: Figure S6 — Parp1 deficiency protects against alkylation-induced pancreatic β-cell death AagTg mice. H&E stained slides of pancreatic β-islets from WT, Aag −/−, Parp1 −/−, AagTg, and AagTg/Parp1 −/− mice either in untreated conditions or 24 h following MMS treatment (150 mg/kg). Representative images are shown of n>2 experiments. The pancreatic β-islets are centered in image and surrounded by pancreatic acinar cells. Untreated sections show healthy pancreatic histology. Following MMS treatment, only AagTg exhibit evidence of pancreatic β-cell toxicity, as illustrated by pyknotic and fragmented nuclei (yellow arrow). Very few intact nuclei are observed in the pancreatic β-islet of the MMS-treated AagTg mice (green arrows). Magnification is 60×; scale bar is 16 µm. (TIF) [file pgen.1003413.s006.tif]

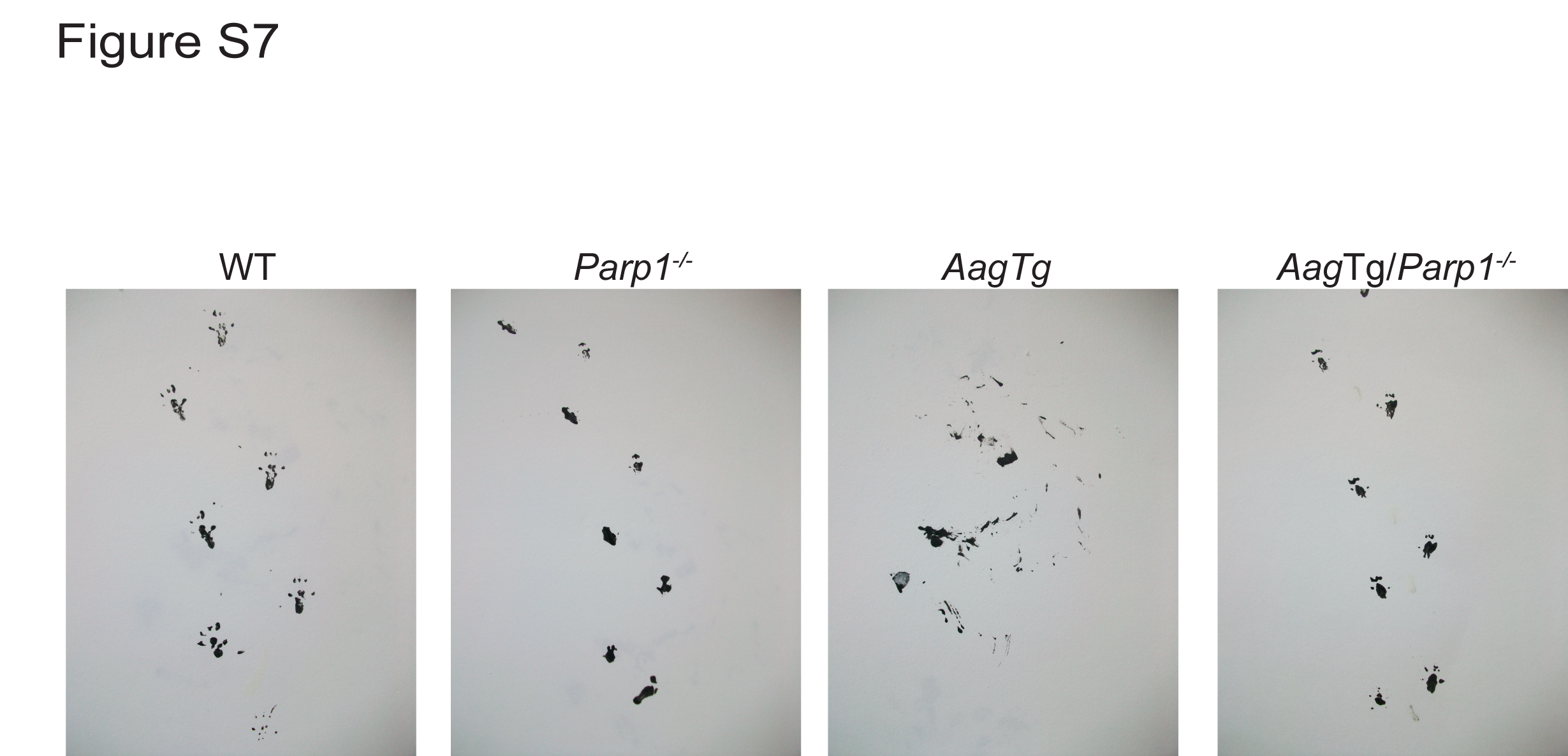

Supplement: Figure S7 — Parp1 deficiency protects against alkylation-induced gait abnormalities. Representations of gait are shown for WT (n = 3), Parp1 −/− (n = 2), AagTg (n = 3) and AagTg/Parp1 −/− (n = 2) mice shown three hours following MMS treatment (90 mg/kg). (TIF) [file pgen.1003413.s007.tif]
