# Supplementary material for: Aag DNA Glycosylase Promotes Alkylation-Induced Tissue Damage Mediated by Parp1
Source: PLoS Genet. 2013 Apr 4;9(4):e1003413. doi: 10.1371/journal.pgen.1003413 (PMC3617098; doi:10.1371/journal.pgen.1003413)
Supplement: Methods S1 — Supplemental Materials and Methods. (DOCX) [file pgen.1003413.s008.docx]

**Methods S1**

**Mice**

*AagTg* mice were generated by injection into FVB/N or C57BL/6 pronuclei using standard procedures by the Department of Comparative Medicine (MIT). Founder line (Fo) 243 was generated using C57BL/6 pronuclear injection (isolated from *Aag^-/-^* mice), whereas Fo 943 and Fo 8756 were derived from FVB/N pronuclear injection. Mice derived from FVB/N pronuclei were backcrossed for 10+ generations before analysis. All transgenic mice were bred to an *Aag^-/-^* background to eliminate confounding results from wildtype Aag activity; *Aag^-/-^*/*AagTg* mice will be referred to in this manuscript as *AagTg* mice. For all experiments examining WT, *Aag^-/-^* and *AagTg* genotypes, mice are >10 C57BL/6 backcross equivalents. However, for experiments examining Parp1 deficiency, the following genotypes were generated on a 129S:C57BL/6 mixed background: WT, *Aag^-/-^, AagTg, Parp^-/-^, AagTg/Parp1^-/-^*.

### Tissue Collection

For aging and survival studies, mice were monitored regularly and euthanized if moribund, or if exhibited decreased body composition. A panel of tissues (heart, lung, kidney, salivary gland, thymus, pancreas, small intestine, spleen, liver, reproductive organs, femurs, brain, eyes, and any tumors) was harvested from every animal and a portion of each tissue were flash-frozen in liquid nitrogen and another portion fixed in formalin or Bouin’s fixative for 18-24 hours and transferred to 70% ethanol.
