# Supplementary material for: Aag DNA Glycosylase Promotes Alkylation-Induced Tissue Damage Mediated by Parp1
Source: PLoS Genet. 2013 Apr 4;9(4):e1003413. doi: 10.1371/journal.pgen.1003413 (PMC3617098; doi:10.1371/journal.pgen.1003413)
Supplement: Table S1 — Aag activity in Aag transgenic founder lines. (DOCX) [file pgen.1003413.s009.docx]

| Table S1. Aag activity in *Aag* transgenic founder lines^#^ | | | | |
| --- | --- | --- | --- | --- |
|  | Genotype | | | |
| Tissue | *Aag*^-/-^ | *Aag* Fo 243* | *Aag* Fo 8756* | *Aag* Fo 943* |
| Bone marrow | 0.19 | 3.29 | 0.06 | 1.24 |
| Brain: cerebellum | 0.22 | 7.95 | 3.83 | 3.24 |
| Brain: cortex | 0.31 | 8.42 | 0.78 | 4.11 |
| Colon | 0.21 | 4.70 | 1.77 | 2.17 |
| Kidney | 0.31 | 5.61 | 0.20 | 2.32 |
| Liver | 0.24 | 8.17 | 0.34 | 1.15 |
| Lung | 0.38 | 7.54 | 0.19 | 3.48 |
| Pancreas | 0.25 | 9.40 | 0.17 | 6.08 |
| Retina | 0.30 | 4.95 | 0.31 | 3.90 |
| Spleen | 0.22 | 4.62 | 0.16 | 2.18 |
| Testis | 0.30 | 1.32 | 0.35 | 1.70 |
| Thymus | 0.14 | 2.86 | 0.25 | 1.19 |

# Aag activity measured by cleavage of hypoxanthine from an oligonucleotide DNA

substrate, and Aag activity is expressed relative to that in WT, which is normalized to 1.

* The *Aag* transgene is expressed in an *Aag^-/-^* background.
